# Usefulness of the Geriatric Nutritional Risk Index (GNRI) as a Predictor of Postoperative Complications After Colorectal Cancer Surgery

**DOI:** 10.7759/cureus.86268

**Published:** 2025-06-18

**Authors:** Yuya Nakamura, Takao Nishimura, Eisho Kanemitsu, Hiromitsu Nagata, Junji Komori, Yasutsugu Takada

**Affiliations:** 1 Department of Surgery, Japan Community Health Care Organization (JCHO) Yamatokoriyama Hospital, Yamatokoriyama, JPN

**Keywords:** colorectal cancer, geriatric nutritional risk index, nutritional risk, postoperative complications, postoperative delirium, postoperative ileus

## Abstract

Introduction: Postoperative complications following colorectal cancer (CRC) surgery are known to not only impair patients' quality of life but also adversely affect long-term prognosis. Objective and simple preoperative risk assessment tools are essential for preventing such complications. The Geriatric Nutritional Risk Index (GNRI), which incorporates serum albumin concentration and the ratio of current to ideal body weight, has emerged as a useful tool for estimating the risk of postoperative complications and long-term prognosis in patients with CRC. However, detailed investigations into its association with specific postoperative complications remain limited.

Methods: We retrospectively analyzed 105 consecutive patients who underwent CRC surgery between 2021 and 2023. Preoperative GNRI was calculated for each patient, and its association with postoperative complications was assessed. For comparison, the Prognostic Nutritional Index (PNI) and the Modified Glasgow Prognostic Score (mGPS) were also evaluated for postoperative complications.

Results: Receiver operating characteristic (ROC) curve analysis identified a GNRI cutoff value of 94 for predicting complications of Clavien-Dindo grade ≥2. Patients in the high-risk group (GNRI <94) had a significantly higher incidence of grade ≥2 complications compared to the low-risk group (14/35 (40%) vs. 14/70 (20%); p=0.029). In multivariable analysis, after adjusting for known risk factors (age, sex, American Society of Anesthesiologists Physical Status (ASA-PS), preoperative obstruction, emergency surgery, surgical approach, and tumor stage), GNRI remained the only significant independent predictor (odds ratio 2.90; p=0.049). While rates of anastomotic leakage and wound infection did not differ significantly between groups, postoperative ileus (6/35 (17%) vs. 1/70 (1.4%); p=0.002) and delirium (4/35 (11%) vs. 1/70 (1.4%); p=0.023) were significantly more frequent in the high-risk group.

Conclusion: GNRI is a simple and effective tool for predicting postoperative complications in CRC patients. Preoperative nutritional assessment using GNRI may enhance perioperative safety and enable more personalized surgical care.

## Introduction

Despite advances in modern surgical techniques, including laparoscopic and robotic approaches, the prevention of postoperative complications remains a major challenge for surgeons. It has been reported that postoperative complications occur in 20-30% of patients undergoing surgery for colorectal cancer (CRC) [1,2]. These complications not only impair patients' quality of life but also adversely affect long-term prognosis [3].

In Japan, the aging population has led to a growing number of elderly CRC patients [4]. Although advanced age alone is not directly associated with a higher incidence of postoperative complications, elderly individuals are generally at increased risk due to age-related declines in organ function and the presence of multiple comorbidities [5]. Various strategies have been introduced to reduce postoperative complications in CRC, including preoperative management of comorbid conditions, minimally invasive surgical techniques, and prehabilitation programs. However, the effectiveness of uniform interventions remains controversial, and there is growing recognition of the need for individualized perioperative care, particularly for patients identified as high-risk [6].

The Geriatric Nutritional Risk Index (GNRI) is a recently developed, simple, and objective tool for assessing nutritional risk in elderly individuals, calculated from serum albumin levels and the ratio of current to ideal body weight [7]. GNRI has been shown to correlate with both short- and long-term outcomes in various clinical settings [8-10]. While previous studies have demonstrated the utility of GNRI in predicting long-term prognosis in CRC patients, few have investigated its association with postoperative complications in detail, particularly with respect to the specific types of complications that may arise [8,11-13].

This study aimed to examine the association between GNRI and postoperative complications in patients undergoing surgery for CRC and to explore the potential utility of preoperative interventions for those identified as high-risk based on GNRI.

## Materials and methods

Study population

This retrospective study analyzed data from patients who underwent surgery for colorectal adenocarcinoma at the Department of Surgery, Japan Community Health Care Organization (JCHO) Yamatokoriyama Hospital, Yamatokoriyama, Japan, between 2021 and 2023. All consecutive cases were considered eligible, while those with missing clinical data were excluded. The study cohort encompassed a wide age range, including both older and younger individuals.

No a priori sample size calculation was performed, as this study utilized all available data from the hospital records during the specified period. The study period was selected to include the most recent three years for which complete data were accessible at the time of analysis.

The study protocol adhered to the ethical standards of the Declaration of Helsinki and received approval from the Institutional Review Board of JCHO Yamatokoriyama Hospital (approval number: R06-1021001). Informed consent was waived due to the retrospective study design, but information regarding the opt-out option was made available on the hospital's website.

Extraction of clinical data, GNRI assessment, and definition of prognostic cutoff value

Relevant clinical data were retrospectively obtained from patient medical records. The clinical data were manually extracted from the hospital's electronic medical record system by the investigators. The extracted variables include body mass index (BMI), sex, age, American Society of Anesthesiologists Physical Status (ASA-PS), primary tumor site (colon or rectum), tumor size, preoperative bowel obstruction (present or absent), surgical approach (open or laparoscopic), type of surgery (elective or emergency), pathological tumor-node-metastasis (TNM) stage (Union for International Cancer Control (UICC) 8th), tumor differentiation (differentiated defined as tubular and papillary adenocarcinoma and others defined as poorly differentiated, mucinous, and signet-ring cell carcinoma), and the presence of postoperative complications. Laboratory data were obtained at the time of the initial visit. GNRI and other nutritional or inflammatory indices were computed as follows.

The GNRI was determined using the formula GNRI=(1.489×serum albumin concentration (g/L))+(41.7×present body weight/ideal body weight (kg)) [7]. Ideal body weight was calculated based on height, assuming a BMI of 22, a method validated in prior studies, instead of the Lorentz formula originally used in the GNRI development [14]. Prognostic nutritional index (PNI) was calculated using the formula PNI=serum albumin (g/L)+0.005×total lymphocyte count [15].

Since GNRI thresholds vary across conditions, we selected a cutoff value of 94 as the optimal threshold for predicting postoperative complications in CRC patients, according to the receiver operating characteristic (ROC) curve drawn with postoperative complications of the Clavien-Dindo classification grade 2 or higher as the outcome (low risk: >94; high risk: ≤94). Patients were considered malnourished when their GNRI was less than or equal to 94.

For the PNI, we referred to previous research that established cutoff values for predicting postoperative complications in CRC surgery [15]. Accordingly, a PNI of 40 or below was considered indicative of malnutrition. Modified Glasgow Prognostic Score (mGPS) was assigned as follows: score 0 for C-reactive protein (CRP) ≤0.5 mg/dL and albumin≥3.5 g/dL; score 1 for CRP >0.5mg/dL or albumin <3.5 g/dL; and score 2 for CRP >0.5 mg/dL combined with albumin <3.5 g/dL [16,17].

These indices are previously validated and widely used in clinical research. No permissions were required for their use in this academic, non-commercial context.

Postoperative complications

Postoperative complications were defined as adverse events occurring during the postoperative hospital stay or within 30 days after surgery. Complications were graded according to the Clavien-Dindo classification. Complications of grade 2 or higher occurring within the defined period were prospectively recorded, evaluated by a clinician, and entered into the clinical database.

Statistical methods

Categorical variables were compared using Pearson's chi-squared test, while continuous variables were analyzed using the Wilcoxon rank-sum test to assess differences between groups. Continuous data are expressed as medians with interquartile ranges.

Univariable logistic regression analysis was performed to identify factors associated with postoperative complications. Variables identified from prior studies as potential confounders were included in the multivariable logistic regression analysis [2,18-21].

A p-value of less than 0.05 on two-tailed tests was regarded as statistically significant. Odds ratios along with 95% confidence intervals (CIs) were computed. All statistical analyses were conducted using JMP Version 18 (SAS Institute Japan Ltd., Tokyo, Japan).

## Results

Characteristics of the study cohort

**Figure 1:**
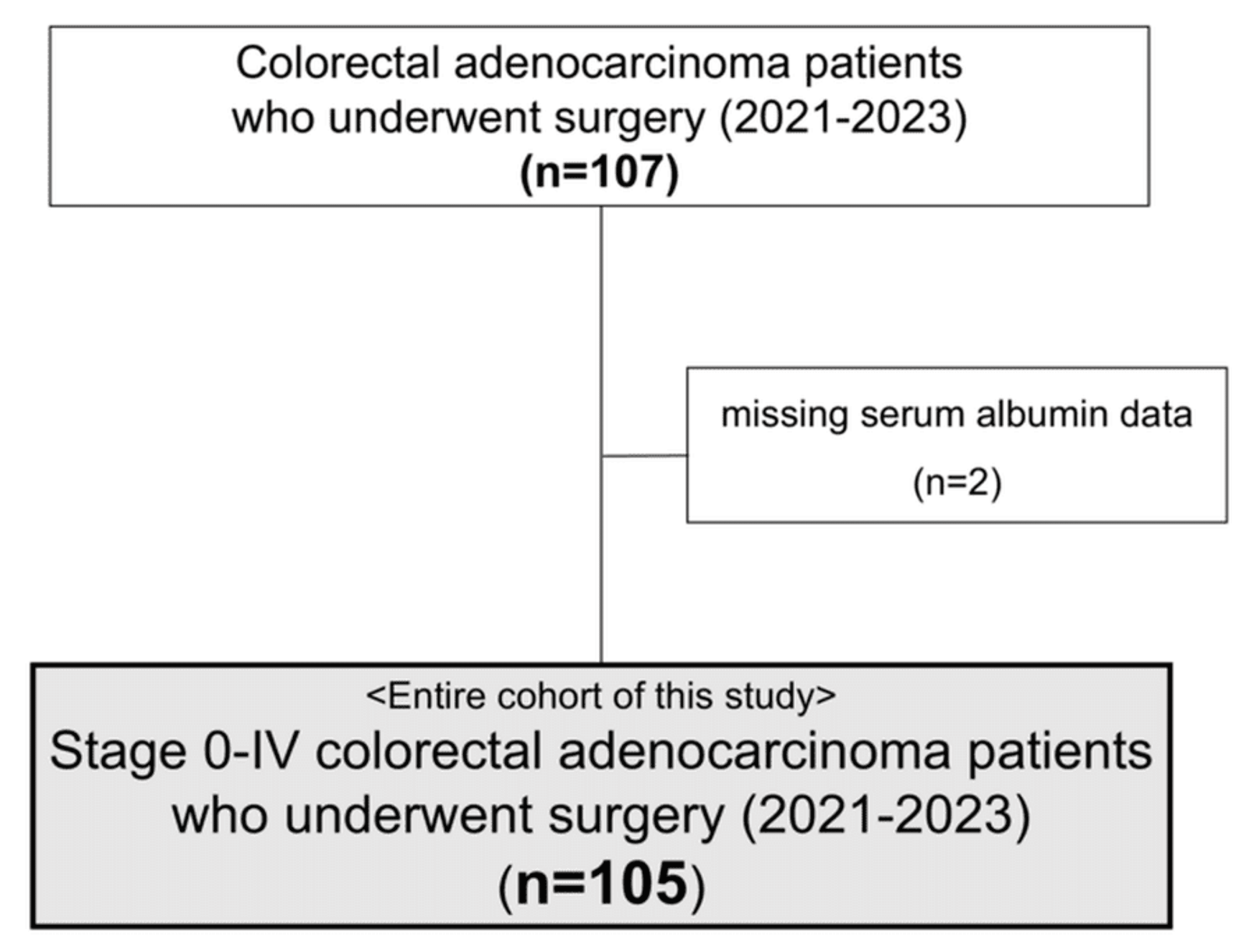
Patient flow diagram of the study cohort. After excluding two patients with missing albumin data from the initially enrolled 107 colorectal cancer surgery patients, a total of 105 patients were included in the final analysis.

Initially, 107 patients diagnosed with CRC and treated surgically at our institution were identified. After excluding two patients due to missing serum albumin data, 105 patients (54 males and 51 females) were included in the analysis (Figure 1).

Of these, 35 patients (33.3%) were classified as having a low GNRI (≤94), while 70 patients (66.7%) had a high GNRI (>94). Table 1 summarizes the clinicopathologic characteristics stratified by GNRI group. There were no significant differences between the groups in term of sex (p=0.679), ASA-PS (p=0.099), primary tumor site (p=0.190), type of surgery (p=0.071), pathological T category (p=0.105), pathological N category (p=0.461), pathological TNM stage (p=0.592), or tumor differentiation (p=0.240). Compared with patients in the high GNRI group, those in the low GNRI group were significantly older (p=0.016), had a lower BMI (p<0.001), had larger tumor sizes (p<0.001), had a higher incidence of preoperative bowel obstruction (p=0.042), and were more frequently treated with open surgery (p<0.001). Additionally, the low GNRI exhibited significantly elevated levels of inflammatory markers (all p<0.001). 

**Table 1:** Patient characteristics and associations of GNRI with clinicopathological factors and inflammatory markers ASA-PS: American Society of Anesthesiologists Physical Status; BMI: body mass index; GNRI: Geriatric Nutritional Risk Index; IQR: interquartile range; mGPS: Modified Glasgow Prognostic Score; PNI: Prognostic Nutritional Index; TNM: tumor-node-metastasis; UICC: Union for International Cancer Control Data are presented as numbers (percentages) or medians (interquartile range). Statistical analyses were performed using Pearson's chi-squared test for categorical variables and the Wilcoxon rank-sum test for continuous variables. The corresponding test statistics (Z or χ²) are presented. A p-value of <0.05 was considered statistically significant, indicated by the asterisk (*).

	Total	Low GNRI (n=35)	High GNRI (n= 70)	Test statistic	P-value
Age, years, median (IQR)	74 (70, 80)	78 (71, 85)	73 (67, 79)	Z=2.40	0.016*
Sex					
Male	54 (51)	17 (49)	37 (53)	χ^２^=0.172	0.679
Female	51 (49)	18 (51)	33 (47)		
BMI, median (IQR)	22.6 (20.2, 25.6)	20.3 (18.1, 21.8)	24.1 (22.4, 27.0)	Z=-5.79	<0.001*
ASA-PS					
1-2	87 (83)	26 (74)	61 (87)	χ^２^=2.71	0.099
≥3	18 (17)	9 (26)	9 (13)		
Primary tumor site					
Colon	88 (84)	27 (77)	61 (87)	χ^２^=1.72	0.190
Rectum	17 (16)	8 (23)	9 (13)		
Tumor size					
<50 mm	42 (40)	6 (17)	36 (51)	χ^２^=11.4	<0.001*
≥50 mm	63 (60)	29 (83)	34 (49)		
Preoperative bowel obstruction					
Present	14 (13)	8 (23)	6 (9)	χ^２^=4.12	0.042*
Absent	91 (87)	27 (77)	64 (91)		
Surgical approach					
Open	15 (14)	12 (34)	3 (4)	χ^２^=17.2	<0.001*
Lap	90 (86)	23 (66)	67 (96)		
Type of surgery					
Elective	101 (96)	32 (91)	69 (99)	χ^２^=3.25	0.071
Emergency	4 (4)	3 (9)	1 (1)		
Pathological T category					
Tis-2	25 (24)	5 (14)	20 (29)	χ^２^=2.63	0.105
T34	80 (76)	30 (86)	50 (71)		
Pathological N category					
N0	71 (68)	22 (63)	49 (70)	χ^２^=0.544	0.461
N1-2	34 (32)	13 (37)	21 (30)		
TNM stage (UICC 8th)					
0	5 (5)	1 (3)	4 (6)	χ^２^=2.79	0.592
I	19 (18)	4 (11)	15 (21)		
II	40 (38)	16 (46)	24 (34)		
III	27 (26)	10 (29)	17 (24)		
IV	14 (13)	4 (11)	10 (14)		
Tumor differentiation					
Differentiated	95 (90)	30 (86)	65 (93)	χ^２^=1.38	0.240
Others	10(10)	5 (14)	5 (7)		
Postoperative complications (Clavien-Dindo grade ≥2)					
Absent	77 (73)	21 (60)	56 (80)	χ^２^=4.77	0.029*
Present	28 (27)	14 (40)	14 (20)		
GNRI, median (IQR)	99.9 (90.8, 108.9)	86.9 (83.1, 90.8)	105.2 (99.8, 111.3)	Z=-8.32	<0.001*
PNI, median (IQR)	45.9 (41.8, 50.8)	39.1 (35.4, 43.0)	48.6 (44.9, 52.5)	Z=-6.48	<0.001*
mGPS					
0	55 (52)	5 (14)	50 (71)	χ^２^=36.6	<0.001*
1	36 (34)	19 (54)	17 (24)		
2	13 (12)	11 (31)	2 (3)		
NA	1 (1)	0	1 (1)		

Risk factors for postoperative complications

Univariable and multivariable analyses of risk factors for postoperative complications are presented in Table 2. In univariable analysis, low GNRI was the only factor significantly associated with increased postoperative complications. Given the association between GNRI and various clinicopathologic factors, multivariable logistic regression analysis was performed. Although some covariates were not statistically significant in univariable analysis, they were included in the multivariable model based on prior literature and clinical relevance. After controlling for known risk factors, including age, sex, ASA-PS, bowel obstruction, surgical approach, type of surgery, and TNM stage, low GNRI remained an independent predictor of postoperative complications (odds ratio range 1.00-8.39) (Table 2).

**Table 2:** Univariable and multivariable analyses of risk factors for postoperative complications ASA-PS: American Society of Anesthesiologists Physical Status; CI: confidence interval; GNRI: Geriatric Nutritional Risk Index; OR: odds ratio; TNM: tumor-node-metastasis; UICC: Union for International Cancer Control; mGPS: Modified Glasgow Prognostic Score ORs are presented with 95% CIs, test statistics (Wald χ²), and p-values. A p-value of <0.05 was considered statistically significant, indicated by the asterisk (*).

Variable	No. of patients	Complication present (N=28)	Complication absent (N=77)	Test statistic	P-value	OR	95% CI	Test statistic	P-value
Age, years									
<80	78	18 (23)	60 (77)	χ^２^=1.99	0.157	Reference			
≥80	27	10 (37)	17 (63)			1.90	0.67-5.43	χ^２^=1.44	0.23
Sex									
Male	54	16 (30)	38 (70)	χ^２^=0.499	0.480	1.74	0.67-4.52	χ^２^=1.30	0.255
Female	51	12 (24)	39 (76)			Reference			
ASA-PS									
1-2	87	22 (25)	65 (75)	χ^２^=0.494	0.482	Reference			
≥3	18	6 (33)	12 (67)			1.32	0.37-4.63	χ^２^=0.19	0.667
Bowel obstruction									
Present	14	2 (14)	12 (86)	χ^２^=1.27	0.261	0.27	0.05-1.47	χ^２^=2.29	0.130
Absent	91	26 (29)	65 (71)			Reference			
Primary tumor site									
Colon	88	21 (24)	67 (76)	χ^２^=2.18	0.140	-	-	-	-
Rectum	17	7 (41)	10 (59)			-	-	-	-
Surgical approach									
Open	15	5 (33)	10 (67)	χ^２^=0.398	0.528	0.61	0.14-2.74	χ^２^=0.41	0.521
Lap	90	23 (26)	67 (74)			Reference			
Type of surgery									
Elective	101	26 (26)	75 (74)	χ^２^=1.16	0.282	Reference			
Emergency	4	2 (50)	2 (50)			2.24	0.22-22.5	χ^２^=0.47	0.495
TNM stage									
0-I	24	4 (17)	20 (83)	χ^２^=2.17	0.337	Reference			
II, III	67	21 (31)	46 (69)			2.87	0.77-10.7	χ^２^=1.47	0.116
IV	14	3 (21)	11 (79)			1.95	0.32-12.0	χ^２^=0.03	0.472
GNRI									
Low (≤94)	35	14 (40)	21 (60)	χ^２^=4.77	0.029*	2.90	1.00-8.39	χ^２^=3.86	0.049*
High (>94)	70	14 (20)	56 (80)			Reference			
PNI									
Low (≤40)	20	7 (35)	13 (65)	χ^２^=1.52	0.468	-	-	-	-
High (>40)	83	21 (25)	62 (75)			-	-	-	-
mGPS									
0	55	12 (22)	43 (78)	χ^２^=2.75	0.431	-	-	-	-
1	36	13 (36)	23 (64)			-	-	-	-
2	13	3 (23)	10 (77)			-	-	-	-

Postoperative outcomes and details of postoperative complications

Postoperative outcomes are summarized in Table 3. The median length of stay was significantly longer in the low GNRI group than in the high GNRI group (median 21 days (range 15-47) vs. 16 days (range 13-22), P=0.021). The incidence of Clavien-Dindo grade II or higher complications was also significantly greater in the low GNRI group compared to the high GNRI group (14/35 [40%] vs. 14/70 [20%], p=0.029). We further performed a stratified analysis based on the surgical approach. Among patients undergoing laparoscopic surgery, the complication rate was significantly higher in the low GNRI group compared to the high GNRI group (43.5% vs. 19.4%, P = 0.022). However, in the open surgery group, no significant difference in complication rates was observed between the two GNRI groups (33.3% vs. 33.3%, P = 1.000). Although both surgical (10/35 [29%] vs. 11/70 [15%], P=0.121) and non-surgical (6/35 [17%] vs.6/70 [8%], P=0.193) complication rates tended to be higher in the low GNRI group, these differences did not reach statistical significance. There was no difference in 90-day mortality between the groups. Similarly, the rates of wound infection and anastomotic leakage did not differ significantly. However, the incidence of postoperative ileus (6/35 [17%] vs. 1/70 [14%], P=0.002) and delirium (4/35 [11%] vs. 1/70 [1.4%], P=0.023) was significantly higher in the low GNRI group than in the high GNRI group (Table 3).

**Table 3:** Postoperative outcomes CD: Clavien-Dindo classification; IQR: interquartile range Data are presented as numbers (percentages) or medians (interquartile range). Statistical analyses were performed using Pearson's chi-squared test for categorical variables and the Wilcoxon rank-sum test for continuous variables. The corresponding test statistics (Z or χ²) are presented. A p-value of <0.05 was considered statistically significant, indicated by the asterisk (*).

Variable		Low GNRI (n=35)	High GNRI (n=70)	Test statistic	P-value
Length of stay, days, median (IQR)		21 (15, 47)	16 (13, 22)	Z=2.30	0.021*
Postoperative complications (CD ≥II), n (%)		14 (40)	14 (20)	χ^２^=4.77	0.029*
Surgical complications		10 (29)	11 (15)	χ^２^=2.41	0.121
Wound infection		2 (5.7)	2 (2.9)	χ^２^=0.520	0.471
Postoperative ileus		6 (17.1)	1 (1.4)	χ^２^=9.26	0.002*
Anastomotic leakage		1 (2.8)	4 (5.7)	χ^２^=0.420	0.517
Abdominal abscess		0	2 (2.9)	χ^２^=1.02	0.313
Lymphorrhea		1 (2.9)	0	χ^２^=2.02	0.155
Hemorrhage		1 (2.9)	0	χ^２^=2.02	0.155
Perforation		0	1 (1.4)	χ^２^=0.505	0.477
Non-surgical complications		6 (17)	6 (8)	χ^２^=1.69	0.193
Pneumonia		0	1 (1.4)	χ^２^=0.505	0.477
Delirium		4 (11)	1 (1.4)	χ^２^=5.15	0.023*
Urinary tract infection		3 (8.6)	2 (2.9)	χ^２^=1.68	0.195
90-day mortality, n (%)		1 (3)	0 (0)	χ^２^=2.02	0.155

## Discussion

Our findings demonstrate that the preoperative GNRI is a useful predictor of postoperative complications in CRC surgery. Patients in the low GNRI group experienced not only a higher complication rate but also significantly longer hospital stays. Notably, detailed analysis revealed a significantly higher incidence of postoperative ileus and delirium in patients with low GNRI.

Of the four previous studies that examined the relationship between GNRI and postoperative complications following CRC surgery, only three provided details on complication types (Table 4) [8,11-13]. These studies consistently reported a higher incidence of infectious complications, including surgical site infections (SSIs), anastomotic leakage, pneumonia, and urinary tract infection, in patients with low GNRI. In contrast, our study did not find a significant difference in infectious complication rates between GNRI groups. One possible explanation lies in the surgical approach: two of the three prior studies reported relatively low rates of laparoscopic surgery (358/1206 (29.6%) and 25/61 (41%)), while the third did not specify. In our cohort, 85.7% (90/105) of patients underwent laparoscopic surgery, which may have contributed to the overall lower infection rates observed (Table 4) [22].

**Table 4:** GNRI and postoperative risk: overview of studies GNRI: Geriatric Nutritional Risk Index; NA: not available; SSI: surgical site infection; UTI: urinary tract infection

Year	Country	Author	N	Complications more common in low GNRI	Rate of lap (%)	Ileus (%) low GNRI vs. high GNRI	Delirium (%)
2020	China	Tang et al. [11]	230	NA	46%	NA	NA
2020	Japan	Sasaki et al. [8]	313	SSI	NA	2.9% vs. 2.3% (grade ≥2)	NA
2021	Taiwan	Liao et al. [12]	1206	SSI, wound dehiscence, UTI, pneumonia	29.6%	5.3% vs. 3.2% (grade ≥2)	NA
2023	Japan	Sato et al. [13]	61	Anastomotic leakage, infectious complication	41%	13% (all grades)	NA
2025	Japan	Our case	105	Ileus, delirium	85.7%	17% vs. 1.4% (grade ≥2)	11% vs. 1.4% (grade ≥2)

Interestingly, we identified postoperative ileus as significantly more common in the low GNRI group, a finding not previously reported. While a high BMI has been identified as a risk factor for postoperative ileus in laparoscopic colorectal surgery, low BMI has also been associated with increased risk [23,24]. One proposed mechanism involves postoperative subcutaneous emphysema in patients with low BMI, leading to hypercapnia. This, in turn, may promote peripheral vasoconstriction and tissue ischemia, predisposing patients to postoperative ileus [24]. Although insufflation pressure and duration were not assessed in our study, these intraoperative factors may influence the risk of postoperative ileus and warrant further investigation, especially in nutritionally vulnerable patients.

Postoperative delirium is a well-known complication, occurring in up to 15-25% after major elective surgery. It is associated with increased mortality, prolonged hospitalization, and long-term cognitive decline [25]. The incidence of CRC surgery ranges from 8% to 54%, highlighting the need for effective risk stratification and prevention [26]. Consistent with previous research in gastric and orthopedic surgeries, our study supports the role of low GNRI as a predictor of postoperative delirium [27,28]. Importantly, nutritional intervention prior to surgery has been shown to reduce the risk of delirium, underscoring the clinical utility of GNRI in perioperative planning.

This study has several limitations. First, although our cohort consisted of consecutive patients, selection bias may exist due to the retrospective design. Second, the relatively small sample size may have limited the power to detect predictive factors beyond GNRI, increasing the risk of type II error. Some established risk factors did not reach statistical significance, possibly reflecting insufficient power rather than a true lack of association. Thus, our findings should be interpreted cautiously and require validation in larger prospective cohorts. Third, although the GNRI cutoff value of 94 was determined by ROC curve analysis in our cohort, it has not been externally validated. Previous studies in elderly or CRC patients have reported similar cutoffs (92-98) [7], but further validation is needed.

Since our study included both elderly and younger patients, it is important to note that GNRI was originally developed for elderly patients by modifying the Nutritional Risk Index (NRI). However, its components, namely, serum albumin and weight status, are fundamental nutritional indicators regardless of age. While our prior studies have shown GNRI's predictive value in younger CRC patients [9,10], its applicability to non-elderly populations requires further study. Lastly, serum albumin levels used for GNRI calculation were obtained at initial presentation, but data on preoperative nutritional interventions were unavailable. This limitation should be addressed in future research.

Notwithstanding the limitations above, our study demonstrates that GNRI is a practical and informative tool for preoperative risk stratification in CRC patients. Notably, it can help identify patients at elevated risk for specific complications, such as postoperative ileus and delirium, for which targeted preventive strategies may be particularly beneficial. These findings contribute to the growing body of evidence supporting individualized, nutrition-informed perioperative care.

## Conclusions

The GNRI is a simple and effective predictor of postoperative complications in CRC surgery patients. Our study showed that low GNRI is significantly associated with higher complication rates, independent of other risk factors. Because GNRI uses routinely measured variables, it can be easily implemented preoperatively to identify at-risk patients. Early nutritional intervention based on GNRI assessment may help reduce postoperative morbidity and improve surgical outcomes.
